# Supplementary material for: Are carbon emissions trading and green financial instruments synergistic? -Comprehensive quantitative research based on content analysis
Source: PLoS One. 2024 Mar 7;19(3):e0298601. doi: 10.1371/journal.pone.0298601 (PMC10919601; doi:10.1371/journal.pone.0298601)
Supplement: S1 Data — (ZIP) [file pone.0298601.s001.zip › Data/地级市Z_F工作报告.zip!/╔╜╢1⁄2/║╩╘≤╩╨/└┤╫╘╣1⁄2╓┌║┼í╛┬φ┐╦╩2╛▌═°í┐_2010.docx]

各位代表:

　　现在,我代表市人民政府，向大会作政府工作报告，请予审议，并请市政协委员提出意见。

　　一、2009年工作回顾

　　刚刚过去的2009年，是新世纪以来我市经济社会发展最为困难的一年。面对国际金融危机持续蔓延带来的严峻挑战和极其复杂的经济形势，全市人民在市委的坚强领导下，深入学习实践科学发展观，认真贯彻落实党中央、国务院和省委、省政府应对国际金融危机的一系列政策措施，紧紧围绕“四个发展”，坚持“四高”要求，振奋精神、坚定信心，开拓创新、埋头苦干，全面完成了市十七届人大三次会议确定的各项任务。

　　——经济平稳较快增长。预计全年实现地区生产总值953亿元，增长14%以上，增幅高于全省3个百分点；一二三产业同比分别增长2.2%、19%、14.4%；三次产业比重达到21.8:51.6:26.6，二三产业占比提高2个百分点。

　　——经济运行质量明显提高。预计规模以上工业企业实现主营业务收入1800亿元、利税170亿元，分别增长22%和36%。完成地方财政收入60.6亿元，增长19.98%，增幅居全省第一位，其中税收收入占地方财政收入的比重达到78.5%，同比提高1.5个百分点。节能减排取得新进展，万元GDP能耗、化学需氧量和二氧化硫排放量继续下降。

　　——城乡面貌进一步改观。创建成为中国优秀旅游城市和省级园林城市。市区40项重点工程顺利完工，县城建设步伐加快，城镇化水平显著提高。新农村建设取得较快进展。

　　——人民生活持续改善。预计城镇居民人均可支配收入12737元，农民人均纯收入4995元，分别增长10%和9%。年末城乡居民储蓄存款余额达到646.3亿元，比年初增加114.3亿元，创历史最高水平。教育、文化、卫生、体育等各项事业全面发展。

　　一年来，我们主要做了以下工作：

　　（一）着力推进大项目建设，加快“四大基地一大产业”发展。抢抓国家扩大投资、拉动内需政策机遇，先后争取中央投资项目355个、资金4.9亿元、省调控资金14.6亿元，争取中央资金数量位居全省第二位，基本实现开工率、资金配套率和问题整改率“三个百分之百”。紧紧围绕“四大基地一大产业”建设，加大招商引资力度，完善项目推进机制，规范项目建设手续，努力破解资金、用地等各种难题，全市新一轮土地利用总体规划率先通过省政府验收，保障了大项目建设顺利进行。规模以上固定资产投资完成475亿元，增长24.9%，其中千万元以上项目完成投资365亿元，增长22%。东明黄河公路大桥获得国家核准，日照至东明输油管道开工建设，东明石化120万吨高硫重油综合利用项目即将建成，龙堌煤矿、赵楼煤矿、巨野巨润建材、东明洪业化工10万吨环己酮、玉皇化工10万吨顺丁橡胶、开发区玉皇20万吨苯乙烯、德润化工5万吨苯加氢、成武达驰750千伏级变压器、牡丹区海普电器、曹县三利轮胎、单县尚舜化工扩建、郓城恒基工程机械、鄄城上海生物肥业、定陶润鑫化工等246个过亿元大项目竣工投产，郓城洪达化工、东明玉皇化工20万吨聚丙烯、牡丹区镁合金、曹县凯雷圣奥等160个过亿元大项目开工建设。“四大基地一大产业”项目完成投资275亿元，占全市千万元以上项目投资的75.3%。

（二）精心组织工业运行，千方百计保增长。把帮助企业度过难关作为保增长、保就业、保稳定的重要措施来抓，成立了工业经济运行指挥部，建立了联系企业督导员制度，制定了促进中小企业稳定健康发展的11条意见，及时帮助企业解决生产经营中的困难和问题，工业经济实现了快速发展。全市规模以上工业企业达到1779家，新增298家；实现增加值483.2亿元，增长23.4％；工业用电量53.4亿千瓦时，增长19.3%,增幅居全省第三位；纳税过千万元的企业达到41家，其中过亿元的7家。市政府与省农业银行、建设银行、农村信用联社等金融机构签订了战略合作协议，大力推动政银企合作，年末各项贷款余额662.7亿元，比年初增加178亿元，同比多增97.3亿元，是历年来增加最多的一年。曹县巨鑫源食品、曹普工艺分别在澳大利亚和美国成功上市。积极实施工业调整振兴规划，狠抓企业技术改造，实施技改项目529个，预计完成投入140亿元，增长67.3％。加快发展高新技术产业，预计实现产值400亿元，增长30%，牡丹工业园被列入全省第一批生物高技术产业基地。加强企业技术中心建设，省级以上达到22家，新增4家。深入实施质量兴市战略，省级名牌产品达到40个，新增17个。繁荣发展商贸物流、文化旅游等服务业，社会消费品零售总额预计完成480亿元，增长19%；实现旅游总收入27.5亿元，增长36.9%；完成进出口总额12.6亿美元，增长26.6%，是全省唯一一个正增长的市。

　　（三）加大“三农”工作力度，扎实推进新农村建设。认真落实各项强农惠农政策，积极发展农业生产，粮食总产111亿斤，增产11亿斤，创历史最高水平；营造片林22万亩，超计划8万亩；肉类、水产品总产量达到55万吨、13万吨，分别增长4.8%、8%。加快推进农业产业化，规模以上农业龙头企业达到600家，新增60家；农民专业合作经济组织发展到1209个，新增302个。高度重视农产品质量安全，市农产品质量安全检测中心基本建成，全市标准化农业生产基地发展到210万亩，获国家认证的无公害农产品、有机食品、绿色食品达到128个。注重改善农村生产生活条件，闫潭、苏泗庄等6大灌区节水改造工程全面启动，郓城南赵楼水库等水源工程建设进展顺利，农田水利基本建设会战持续开展，完成水利建设投资11.9亿元。农机总动力达到1288万千瓦，农作物耕种收基本实现机械化。改造国省道干线公路200公里、农村公路1720公里，新建电气化村48个、户用沼气池2.4万座，新增饮水安全人口65万人。实施整村住房建设改造160个，完成农房建设5.5万户，改造危房1.8万户。培训农民22万人次，新增农村劳动力转移就业24万人。

（四）强化规划建设管理，完善提升城市功能。组织国内一流的规划设计单位和专家，高水平编制了城北组团、城南组团、西部城区等11项分区规划、控制性详细规划和城市设计，市区控制性详规覆盖率达到100%，提前一年完成了省政府确定的工作目标。县城控制性详规覆盖率达到70%以上。继续实行项目指挥部工作机制，进一步完善联席会议、周调度会等推进制度，市区重点工程明显加快，完成投资70亿元，创历年新高。大剧院、演武楼、牡丹广场等重点工程竣工投用，大剧院片区成为城区新地标。赵王河下游开发治理工程基本完工，沿河景观带初步形成。按照5A级景区标准实施了曹州牡丹园提升改造，园区面积扩大了600亩，土建工程基本完成。南外环路开工建设，和平路、西安路实现贯通，新修、改造市区道路20余条、40多公里。市垃圾处理厂竣工运营，南湖水库一期建成蓄水，两区污水处理厂开工建设，新建雨污管网66公里。铺设热力管道9.6公里、燃气管道12.8公里，新增供热面积120万平方米、天然气用户8000户。栽植各类乔灌木30万棵，新增改造绿地43处。实施了华英路、赵王河景观带、大剧院片区和重要节点的亮化工程，市区亮化水平得到提升。大力推进房地产综合开发，市区完成拆迁面积130万平方米，新开工商品房面积150万平方米，其中高层、小高层115栋，竣工商品房面积120万平方米。加快保障性住房建设，建成廉租住房2万平方米。深入开展了以“迎全运庆国庆共建文明牡丹城”活动为重点的环境综合整治，城市面貌明显改观。县城建设力度进一步加大，新一轮城镇建设全面启动。

（五）加快发展社会事业，努力促进社会和谐。教育事业全面发展，改造农村中小学危房31.5万平方米，新建城区中小学15所；中小学课程改革走在全省前列；菏泽学院规模、质量、结构、效益协调发展，菏泽医专新校区投入使用；职业教育水平进一步提升，菏泽家政职业学院、菏泽技术学院内涵建设、招生就业取得新成效，菏泽职业技术学院筹建工作迈出新步伐。卫生工作得到加强，完成了2332所村卫生室、29个社区卫生服务机构的建设，新农合参合率达到98.9%；手足口病防治工作取得了阶段性胜利，甲型H1N1流感防控科学有效。建立了计划生育利益导向机制，群众生育观念进一步转变，人口自然增长率控制在5.59‰。文化信息资源共享工程扎实推进，新改扩建乡镇文化站46家，新建农家书屋748个，新发展城乡有线电视用户3.5万户。十一届全运会武术散打决赛圆满成功，荣获国家体育总局重大贡献奖和十一届全运会优秀赛区。努力扩大就业，城镇新增就业11万人，登记失业率控制在3.8%；完善社会保障体系，各项社会保险新增参保人口106.8万人，城乡低保标准分别提高到每人每月200元和每人每年1000元，城乡大病人均救助标准分别提高到3800元和2800元，农村“五保”集中供养率达到70%以上，新农保试点工作顺利启动。食品药品监管工作成效明显，安全生产四项指标全面下降，社会治安形势持续稳定。对口援建都坝乡重建工程全部完工，援建北川新县城项目进展顺利。人防、气象、地震、档案、史志、民族宗教、外事侨务、对台工作、民兵预备役、老龄、妇女儿童和残疾人等各项事业都取得了新进展。

（六）加强政府自身建设，不断提高行政效能。坚持向人大报告工作和向政协通报工作制度，自觉接受人大的法律和工作监督、政协的民主监督，认真执行市人大及其常委会决议，积极办理人大代表建议和政协委员提案，办复率达到100%。继续实行服务承诺、限时办结、部门主要负责人电话公开和集中收费等制度，充分发挥市长公开电话、行风热线等便民渠道的作用，解决了一大批群众关心的热点难点问题。精简审批事项，开展行风评议活动，经济发展环境进一步改善。加强行政监察和审计监督，廉政建设取得了新成效。政府机构改革稳步推进。争先创优工作成效显著。

各位代表，在国内外市场低迷、企业运行困难、利润空间下降、投资动力不足、财政收支压力较大的情况下，取得这样的成绩极其不易。这是市委正确领导的结果，是市人大、政协和社会各界有效监督、大力支持的结果,是全市人民和衷共济、扎实苦干的结果。在此,我代表市人民政府,向全市广大干部群众，向各民主党派、工商联、人民团体和社会各界人士,向驻菏部队、武警官兵和中央、省属单位,向所有关心支持菏泽发展的朋友们,表示诚挚的敬意和衷心的感谢!

在充分肯定成绩的同时，我们也清醒地认识到，我市经济社会发展还面临着不少困难和问题，主要是：经济总量小，结构不合理，工业化、城镇化发展滞后，企业市场竞争和自主创新能力弱；国家加大经济结构调整力度，提高市场准入、生产要素供应、节能减排等准入门槛，对我市大项目建设提出了更高要求；农业结构调整进展慢，产业化水平低，农民增收依然困难；财政政策性减收因素多，硬性增支压力大，收支矛盾较为突出；就业形势不容乐观，社会保障能力不强，社会事业发展还不能很好地满足群众需要；政府工作人员的服务质量和效率有待进一步提高，等等。对此，我们将采取有力措施，切实加以解决。

二、2010年目标任务和工作重点

2010年是实施“十一五”规划的最后一年，也是实现2012年奋斗目标、为“十二五”发展打基础的关键一年，做好今年工作至关重要。新的一年，机遇与挑战并存，但机遇大于挑战。国家继续实施积极的财政政策和适度宽松的货币政策，有利于我市大项目建设、中小企业发展和民生的改善；省委、省政府要求我市打造鲁苏豫皖四省交界地区科学发展高地，有利于我市争取上级的政策和支持。新一轮城镇化建设高潮的兴起，不仅能够完善城镇功能，而且能够带动相关产业加快发展；在过去两年竣工投产300多个大项目的基础上，今年新开工和续建大项目还将达到300个；郭屯煤矿可正式投产，郓城煤矿有望试生产，加上已投产的彭庄、龙堌、赵楼煤矿，今年煤炭产量可达到700万吨以上，这将是我市经济发展和财政收入的重要增长点。我们必须进一步坚定信心、抢抓机遇，发挥优势、趋利避害，开拓创新、扎实苦干，把推动经济社会发展的各项工作做得更好。今年政府工作的总体要求是：全面贯彻党的十七大和十七届三中、四中全会精神，以邓小平理论和“三个代表”重要思想为指导，深入学习实践科学发展观，坚持走新型工业化和新型城镇化道路，以转方式、调结构、增效益为主线，以“四大基地一大产业”建设为重点，着力推进大项目建设，着力提升工业运行质量，着力培育现代服务业，着力加快城镇建设，着力发展农业产业化，着力保障和改善民生，推动全市经济社会科学发展、和谐发展、跨越发展、更好更快发展，为打造鲁苏豫皖四省交界地区科学发展高地打下坚实基础。 全市经济社会发展的主要预期目标是：地区生产总值增长15%；规模以上工业增加值增长21%；高新技术产业产值占规模以上工业的比重达到25%；千万元以上项目固定资产投资增长20%；社会消费品零售总额增长18%；外贸进出口增长8%；外商直接投资增长10%；地方财政收入增长16%；城镇居民人均可支配收入和农民人均纯收入均增长9%；居民消费价格总水平控制在3%左右。人口自然增长率控制在7.5‰以内；万元GDP能耗降低4.2%，SO2、COD排放量分别下降2%和3%。

围绕实现上述目标，重点做好以下六项工作:

（一）全力推进大项目建设，实现工业化进程的新跨越

转方式、调结构是中央和省对今年经济工作作出的重大战略部署，也是我市经济工作的主攻方向和重要任务，必须把着力点放在加快推进新型工业化进程上、放在加快大项目建设上，大力开展大项目建设推进年、提升年活动，用大项目建设的大成效、高水平，促进工业经济的大提升、大发展。按照“四个一批”的要求，抓策划、抓招商，促储备；抓审批、抓完善，促规范；抓开工、抓进度，促建设；抓服务、解难题，优环境；抓竣工、抓投产，促成效，确保牡丹区华润电厂，东明石化300万吨高硫重油综合利用、洪业化工16万吨己内酰胺，郓城赵楼煤矸石电厂，巨野巨森秸秆木材综合利用、40万吨醋酸，开发区聚隆化工等一批大项目尽快开工；巨野铁雄新沙二期、单县舜亦新能源一期、曹县凯雷圣奥、郓城洪达化工等一批大项目年内竣工投产，力争全年新开工、续建亿元以上大项目300个以上，完成投资300亿元以上，新增主营业务收入700亿元以上。在推进项目建设工作中，注意把握好以下几点：一是把做大做强企业贯穿大项目建设的全过程。全面实施中小企业成长计划，在立项、技改、融资、科技、服务等方面加大支持力度，利用3年时间实现“一六五五”发展目标：到2012年完成主营业务收入过500亿元的企业1户，过100亿元的6户，过10亿元的50户，过亿元的500户。二是把扩大开放、招商引资贯穿大项目建设的全过程。抓住国家推进产业转移的机遇，充分利用国际牡丹花会和中国林交会两大平台，改进招商引资方式，提高招商引资实效。在各县区分散招商的基础上，市里将统一组织两次以上集中招商活动。全市确保落地20个投资10亿元以上、200个亿元以上的大项目。扩大市场准入范围，进一步激活民间投资。三是把推动传统产业优化升级贯穿大项目建设的全过程。围绕纺织、食品、木材、机械等传统产业优化升级上高水平项目，力争新上项目的设备、工艺和技术达到国内同行业先进水平。四是把精深加工、拉长产业链条贯穿大项目建设的全过程。围绕拉长产业链条，发展精深加工，策划和引进一批大项目、好项目，实现资源就地转化，提高产品附加值，变资源优势为经济优势，促进“四大基地一大产业”向深度、广度、集聚度发展。五是把着力培植战略性新兴产业贯穿大项目建设的全过程。利用研发投入抵扣所得税政策和省设立战略性新兴产业专项资金的机遇，抓紧策划论证相关项目，积极发展新能源、新材料、生物医药等战略性新兴产业。六是把发展低碳经济、推进节能减排贯穿大项目建设的全过程。树立低碳经济理念，坚定不移地走低碳发展之路。严格执行环评和能评制度，坚决杜绝“两高”项目上马。对照国内同行业最高标准，加快企业技术改造，确保达到节能减排要求。抓好污水处理和中水回用工程正常运行，建成市区污水处理厂2座，新建污水管网150公里，确保城市污水集中处理率达到80%，三条主要河流出境断面水质进一步改善。严格土地管理，提高土地节约集约利用水平。

（二）强化工业运行调度，提高发展质量和效益

要克服金融危机带来的不利影响，有效解决经济发展中诸多困难和问题，确保新的一年有一个更好更快的发展，必须下大气力抓好工业经济运行。一是加强组织协调，切实解决实际问题。强化工业运行调度分析，继续坚持工业运行指挥部工作机制，完善重点企业督导员制度，认真落实扶持60户重点企业和鼓励支持“一六五五”企业发展的政策措施，搞好煤电油运综合协调，促进企业快速健康发展。进一步优化金融生态环境，加强政银企合作，继续实行对金融机构的奖励政策，着力解决中小企业融资难，力争全市新增贷款150亿元以上，其中中小企业贷款80亿元以上。努力拓宽融资渠道，力争新增上市企业3家，进入上市程序的15家，并有1家市外金融机构在我市落户。二是大力开拓市场，畅通营销渠道。抓住市场回暖和国家实施扩大消费的政策机遇，从提供市场信息、组织企业参加商品交易会等方面入手，引导企业组织生产适销对路产品，努力保持和提高市场占有率。调整优化出口产品结构，着力推动骨干企业出口，特别是自营出口，确保全市出口情况好于去年。三是切实加强管理，努力降低成本。在全市企业中全面开展“练内功、挖潜力、增效益”活动，进一步推动企业加强财务、生产、现场等基础管理，高度重视解决两项资金占用问题，提高资金流转速度和效率。实施质量兴市和名牌带动战略，工业产品标准覆盖率达到95%以上，力争新创省级以上名牌产品10个。加快推进企业改革，进一步完善企业法人治理结构。四是加大技改力度，提高装备水平。鼓励企业采用高新技术对产品设计、生产制造、市场营销等环节进行改造，加快设备更新，提高工艺装备水平和产品附加值。全市实施技改项目600个，完成投资180亿元以上。推进信息化与工业化融合，支持企业广泛应用信息技术，提升自动化、智能化和管理现代化水平。五是强化自主创新，增强研发能力。重点推进前200家规模以上企业技术中心建设，新增省级以上企业技术中心2家；鼓励企业广泛开展产学研联合，突出抓好10个重点产学研合作项目和10个重大自主创新项目的实施；全年取得重要科技成果100项以上，全社会研发投入占GDP的比重达到0.8%以上。加大人才引进培养力度，全年引进30名高层次职业经理人、30名博士、300名硕士，培养30名以上有一定影响力的专业技术人才。六是加快发展高新技术产业。继续加强菏泽高新技术产业开发区建设，争取年内通过省政府验收；扶持30家重点高新技术企业加快发展，全市完成高新技术产业产值540亿元以上。

（三）大力发展现代服务业，实现结构调整的新突破

现代服务业不仅是工业化、城镇化的强大支撑，也是转方式、调结构的必然选择，必须把服务业发展作为经济工作的一大重点，切实抓紧抓好，力争服务业增加值占GDP的比重比上年提高0.7个百分点以上。一是强化“四大载体”建设。继续加大对20个重点服务业城区、10个重点服务业园区、30个重点服务业企业、50个重点服务业项目的扶持力度，加强调度考核，确保重点城区服务业税收占重点城区工商税收的比重提高3个百分点；重点服务业园区交易额和重点服务业企业营业收入增长20%以上；重点服务业项目完成投资28亿元以上。二是着力发展第三方物流。坚持规划指导、试点先行，总结推广东明石化、巨野巨润建材二三产业分离经营的经验做法，研究制定具体政策措施，加快推进企业二三产业剥离，优化企业资源配置，拓宽服务业增长空间。三是加快商贸物流项目建设。大力推进中国牡丹商贸城、菏泽国贸中心、定陶国大农贸城、单县湖西财富广场、鄄城中药材大市场等项目建设进度，力争年交易额过100亿元的物流园区达到2处以上、过30亿元的市场达到10处以上。四是重点发展文化旅游等新兴服务业。集中抓好郓城水浒文化旅游城、鄄城孙膑旅游城、巨野金山旅游区、成武文亭湖旅游区、单县浮龙湖旅游区、曹县太行堤水库万亩荷塘旅游区等景区开发建设，充分发挥好大剧院、演武楼的作用，加大宣传推介力度，打造“一城四乡一生态”旅游品牌，确保旅游总收入增长30%以上。加快发展会展、金融、保险、法律、会计、电子商务等新兴服务业。五是努力扩大居民消费。认真落实国家扩大消费的各项政策，切实做好家电、摩托车、汽车、农机下乡和家电、汽车以旧换新工作，继续实施“万村千乡”市场工程和“农产品进超市”工程，有效促进城乡居民消费。进一步优化消费环境，严厉打击假冒伪劣、价格欺诈、非法广告等各种不法行为，维护消费者合法权益。

（四）全面实施城镇建设三年纲要，加快推进新型城镇化

　　城镇化是工业化的重要载体，是促进居民消费、调整区域结构的重要切入点。紧紧抓住国家和省推进城镇化机遇，全面实施市、县《城镇建设三年纲要》，提升城镇化发展水平。一是进一步完善城镇规划。有针对性遴选高水平的规划设计单位，高标准做好城市商业金融中心、林展馆片区、东部城区综合区、人民路、黄河路的城市设计，以及城市重点工程的方案与施工图设计。组织各县编制道路交通、绿地水系等专项规划，县城控制性详规覆盖率达到100%，乡镇总体规划全部完成。规范“六线管理”制度，从严管理用地性质和容积率调整。二是大力推进城镇重点基础设施建设。加快德商高速北段工程建设，开工建设东新高速、菏泽火车站改扩建工程，做好菏鱼高速、万福河、郓巨河航道前期工作，力争洙水河年内通航，鄄城黄河公路大桥年底通车。重点抓好11项电网建设项目，完成电网建设投资13亿元。实施南外环及220国道连接线、长城路、大学路、中山路、点将台路等一批道路桥梁工程，加大对老城区道路、背街巷改造力度。启动市图书馆、规划展览馆建设。加强以县城基础设施为重点的城镇建设，增强城镇综合承载能力。三是注重生态环境建设。继续推进赵王河下游开发治理，开工建设生态湿地保护区，力争把赵王河打造成纵贯城市南北、具有现代气息和生态价值的重要景观带和城市防汛主河道。加快曹州牡丹园提升改造工程建设，确保四月上旬开园迎宾。开工建设胜利公园、烈士陵园、洙水河公园、万福河公园，深入实施补树增花规划，统筹建设供水、供热、雨水、污水等管网工程。坚持城镇管理重心下移，全面推行城镇精细化管理，提高净化、亮化、美化水平。再接再厉，积极开展国家园林城市创建工作。四是加快城市综合开发。围绕旧城改造和城中村改造，加大项目策划、招商和建设力度，集中打造一批精品片区，力争全市竣工商品房面积800万平方米，其中市区260万平方米以上。依法严厉打击违法搭建行为，维护好城市开发建设秩序。加强建筑业市场监管和质量安全管理，发展壮大建筑业。五是创新投融资机制。用足用好国家政策，最大限度地争取国债资金和各种专项资金。发挥政府资金的导向作用，吸引社会资金投入城镇建设。努力做大城建投融资平台，力争在利用金融机构贷款上实现大的突破。进一步深化市政公用事业改革，规范特许经营管理，增加经营收益。六是积极做好户籍制度改革工作。按照国家和省里的统一部署，全面放开县域内户口迁移政策，改革户籍管理相关配套制度，使有条件的农村居民有序转移为城镇居民。

（五）深入推进农业产业化经营，促进农业增效农民增收

我市是农业大市，“三农”工作始终是各项工作的重中之重。围绕农业增效、农民增收目标，努力促进农业和农村经济全面发展。一是加大龙头企业培育力度。促进52家支撑企业、30家重点龙头企业快速膨胀，力争新增1家国家级、5家省级、20家市级农业产业化龙头企业。规模以上龙头企业主营业务收入达到870亿元，增长16%以上。加快农产品原料基地建设，力争达到600万亩。积极发展农民专业合作组织，全市新增300家以上。切实抓好优势农产品标准化生产示范县建设，新增无公害农产品、绿色食品、有机食品认证20个以上、标准化生产基地30万亩。二是积极推进农业结构调整。在稳定粮食生产的同时，大力培育大蒜、芦笋、西瓜、甜瓜、山药、牡丹、药材等规模化区域示范基地，新增经济作物面积60万亩以上。深化集体林权制度改革，加强美国白蛾防控，新增造林面积8万亩以上。加快推进规模养殖场区建设和“128渔业示范工程”，新增林下规模养殖场区150个以上，水产品养殖面积达到35万亩。抓好5个“一乡一业”示范乡镇、100个“一村一品”示范村建设。继续实施农业科技示范工程，加强科技特派员工作，力争下派1200人以上。三是努力改善农业生产条件。认真落实党的各项强农惠农政策，及时将各种补贴足额发放到农民手中。继续推进闫潭、谢寨等6大灌区改造，全面完成单县浮岗水库和曹县太行堤水库除险加固，加快郓城南赵楼水库建设，尽快启动南湖水库二期、东明拦蓄工程和牡丹区都司拦蓄工程，深入开展农田水利基本建设会战，及时做好黄河水引蓄调工作，增强综合抗旱防涝能力。强化农村公共基础设施管理，建立健全长效管护机制。大力开展农机规模化作业，农作物综合机械化水平达到77%以上。四是促进农村劳动力转移。加快建立服务、培训、维权三位一体的农村劳动力输出工作机制，广辟就业渠道，引导广大农民外出就业、就地创业。以菏泽技术学院为龙头，健全市县乡三级职业培训网络，力争培训农村劳动力30万人次。坚持依法、自愿、合理的原则，鼓励农民通过转包、出租、股份合作等形式流转土地承包经营权，发展适度规模经营。五是稳步推进新农村建设。坚持与小城镇建设相衔接，加大试点村（中心村）规划和建设力度。建设农村社区300个，社区覆盖率达到30%以上。

（六）切实做好十项重点民生工作，促进社会和谐稳定

保障和改善民生是发展经济的最终目的，也是政府工作的根本出发点和落脚点。为了解决事关群众切身利益的实际问题，保障和改善民生，促进和谐社会建设，今年切实抓好10项重点工作。（1）千方百计扩大就业。以项目带就业，以创业促就业，确保全年新增城镇就业9万人，力争达到10万人以上，登记失业率控制在4%以内；转移农村劳动力18万人以上。（2）进一步提高社会保障水平。加强社会保险扩面征缴，各项社会保险新增参保人口18万人次；城乡低保标准分别提高到每人每月220元和每人每年1200元；城乡大病救助标准分别提高到4000元和3000元；新型农村合作医疗、城镇居民医疗保险补助标准均提高到100元。（3）改善城乡居民生活条件。市区新增集中供热面积210万平方米、天然气用户2万户；新建改造路灯50公里；新增公交线路8条、公交车100辆，缓解城市交通压力。全市新建改造农村公路1000公里以上、窄危桥50座；新建农村户用沼气池2.4万座，新增农村安全饮水人口60万人。（4）加强保障性住房和农村住房建设。全市新建经济适用房3000套、25万平方米，廉租住房2000套、10万平方米；启动整村建设改造315个，力争新建农村住房7万户，确保改造农村危房3.5万户。（5）改善办学条件。加固改造中小学校舍25万平方米以上；各县区至少新建1所城区中小学校；中小学现代远程教育网络实现“校校通”。支持菏泽学院二期工程、菏泽职业技术学院一期工程建设。（6）增强教师队伍素质。全市选拔1000名以上优秀城镇教师到农村对口支教；面向社会公开招录1000名以上优秀毕业生充实教师队伍；选拔1000名菏泽学院优秀师范生到农村学校顶岗实习，被顶岗教师到菏泽学院脱产学习半年。（7）加快医院项目建设。开工建设市中医院和市精神病防治中心项目；完成牡丹区、郓城县人民医院，启动曹县、单县、巨野县、定陶县人民医院标准化建设；完成8处乡镇中心卫生院建设任务。（8）强化公共卫生服务。积极稳妥推进医药卫生体制改革，实施妇幼保健等9项基本公共卫生服务项目，实施重大疾病防控等6项重大公共卫生服务项目，完成15岁以下儿童免费补种乙肝疫苗、10万名农村妇女孕前和孕早期免费补服叶酸，做好10万名农村产妇住院分娩和1500例白内障复明手术补助工作。（9）完善公共文化基础设施。启用鲁西南民俗博物馆；完成单县、定陶县、巨野县“三馆”和全市147个乡镇综合文化站、300个农村文化大院、1000家农家书屋建设。（10）丰富城乡文化生活。举办第三届农村文化艺术节等5大文艺活动，送戏下乡1000场，放映农村公益电影7万场，完成10万户有线电视数字化平移工作。

　　在做好以上10项重点民生工作的同时，进一步加强社会管理，推进平安建设。认真执行领导干部公开接访、约访、下访制度，紧紧抓住影响社会和谐稳定的源头性、根本性、基础性问题，深入推进社会矛盾排查化解。扎实开展普法教育，做好法律援助工作。完善各项应急救援机制，提高抗灾救灾和处置突发事件的能力。继续加强安全生产基层基础工作，着力推进29个安全社区创建；实施科技兴安战略，加快安全科技成果推广应用，完成149家化工企业安全自动化改造；突出抓好重点行业和领域的安全监管，严防事故发生。认真组织开展食品药品安全专项整治活动，严格市场准入，强化责任追究，确保群众饮食用药安全。全面落实社会治安综合治理各项措施，着重强化村居治安巡防工作，建立健全社会治安防控体系，深入开展严打整治斗争，始终保持对刑事犯罪的高压态势，切实增强人民群众的安全感，确保社会长治久安。进一步做好人口计生、民族宗教、外事侨务、体育、档案、史志、人防、地震、气象、对台等工作，促进各项社会事业全面发展。

　　三、进一步加强政府自身建设

　　始终把对人民负责、让人民满意作为政府工作的第一准则、第一追求，以转变政府职能、提高行政效能为核心，加快制度创新和管理创新，切实加强自身建设，努力打造负责任、有作为、人民满意的政府。

　　（一）强化学习意识，提高工作水平。加强学习型机关建设，营造学习氛围，优化知识结构，提高公务员队伍综合素质。深入研究国家宏观经济政策，努力创造更为有利的区域发展环境。注重对经济社会发展全局性、前瞻性、战略性问题的思考，善于运用创新的思维、改革的思路、积极的办法、科学的机制，解决好发展中的难点问题和人民群众关注的热点问题，不断提高推动经济发展、促进社会和谐的水平和能力。

　　（二）强化责任意识，狠抓工作落实。按照干事干事再干事、落实落实再落实的政府工作定位，认真执行好市委确定的七大工作机制，继续推行“一线工作法”，强化工作的交办、催办、督办、查办，让每一项工作、每一个项目、每一件实事都有明确的责任主体、目标时限要求和奖惩考核手段，切实做到定一件、干一件、成一件。牢固树立群众观念和公仆意识，坚持深入实际、深入基层，思想上尊重群众、行动上深入群众、工作上依靠群众，做到问政于民、问需于民、问计于民。大力弘扬求真务实精神，坚决反对形式主义、官僚主义和弄虚作假行为，努力营造重实绩、干实事、求实效的浓厚氛围。

　　（三）强化服务意识，优化发展环境。深化服务承诺、限时办结、部门主要负责人电话公开“三项工作”，不断提高服务质量和办事效率。在所有企业和个体工商户中推行集中缴费，坚决制止乱检查、乱收费。强化审批管理，精简审批事项，优化审批流程，缩短审批时限。深入开展行风评议活动，对行政效能情况实行实时电子监察，促进政风行风进一步好转。充分发挥纠风办、行政效能投诉中心、市长公开电话受理中心等机构的监督职能，及时受理企业和群众投诉，严查各类破坏发展环境案件，坚决纠正损害群众利益的不正之风，倾力打造让企业安心、舒心、顺心的发展环境。

　　（四）强化法制意识，推进依法行政。坚持领导干部学法制度，搞好干部法律知识培训，提高各级政府工作人员依法行政的意识和能力。完善科学民主决策机制，健全重大问题、重点事项的集体决策、专家咨询、社会公示和听证制度，实现行政决策的科学化、民主化。全面推行行政执法责任制，完善执法监督、评议考核、行政赔偿和过错追究制度，切实做到严格、公正、文明执法。自觉接受人大法律和工作监督、政协民主监督、新闻舆论监督和社会公众监督，主动听取各民主党派、工商联、无党派人士和人民团体的意见建议。完善和加强政府内部监督，认真做好政府信息公开工作，深入推进政务公开和公共企事业单位办事公开，增强政府工作透明度。

　　（五）强化廉洁意识，坚持从严治政。我们的政府是人民政府，权力来自于人民。我们将牢记宗旨、忠于使命，廉洁勤政、为民造福，做到反腐倡廉常抓不懈、拒腐防变警钟长鸣。按照“一岗双责”要求，认真落实党风廉政建设责任制。深入开展反腐倡廉教育，筑牢拒腐防变的思想道德防线。坚持用制度管权、管事、管人，健全权力运行制约和监督机制。进一步加强公务用车管理，严禁公车私用。自觉树立过紧日子的思想，强化预算约束和财政管理，带头压缩行政经费支出，规范和减少各类会议、接待等活动，降低行政成本。筹建市公共资源交易大厅，推进政府采购、工程招投标、土地招拍挂、产权交易的阳光操作。加大案件查办力度，严肃查处商业贿赂、重大责任事故、工程建设领域等方面的案件，依法严惩腐败分子。各级政府和各部门主要负责人是廉政建设的第一责任人，要严格遵守廉洁自律各项规定，自觉做反腐倡廉的表率，以实际行动带班子促队伍，推动廉政建设取得新成效。

　　各位代表！我们正处在加快发展、实现赶超的关键时期，新的形势催人奋进，新的任务光荣艰巨。让我们在中共菏泽市委的坚强领导下，解放思想，抢抓机遇，同心同德，以十足的干劲扎实苦干，推动全市经济社会科学发展、和谐发展、跨越发展、更好更快发展，在打造鲁苏豫皖四省交界地区科学发展高地进程中做出新的更大成绩！
